# Supplementary material for: Translating functional brain activity between humans and monkeys
Source: bioRxiv. 2026 Oct 2:2026.10.01.755966. Preprint. [Version 1] doi: 10.64898/2026.10.01.755966 (PMC13648740; doi:10.64898/2026.10.01.755966)
Supplement: Supplementary file 1 [file NIHPP2026.10.01.755966V1-supplement-1.pdf]

bioRxiv preprint doi: https://doi.org/10.64898/2026.10.01.755966; this version posted October 2, 2026. The copyright holder for this preprint (which was not certified by peer review) is the author/funder, who has granted bioRxiv a license to display the preprint in perpetuity. It is made available under a CC-BY 4.0 International license.

SUPPLEMENTARY

Supplementary Discussion

Multiview Temporal Alignment (MVTA). MVTA seeks to learn a convolutional kernel that aligns MION data directly to BOLD. We compared it to several alternatives using simulated data with known ground-truth structure. A central challenge is that observed fMRI signals potentially reflect mixtures of multiple sources of variance (e.g., components shared across species, components shared only within a species, and components unique to individual subjects). Only the shared component is informative for learning cross-species temporal transformations, but unshared components may obscure the shared structure needed to learn a meaningful transform.

We generated five synthetic fMRI runs for two "macaque-like" and two "human-like" datasets, each comprising multivoxel responses driven by multiple latent neural signals.

First, we generated latent neural time series for 250 synthetic voxels from three classes of signals: (i) two signals shared across species ("shared"), (ii) two signals shared only within species ("group-specific"), and (iii) two subject-specific signals. Neural signals ($X_{neural} \in R^{T \times N_{signals}}$) were generated as random binary sequences (Bernoulli process). In real data, the relative contributions of these signal classes are unknown. Our goal was to include sufficient unshared neural variance to mimic the species- and individual-specific representational structure that we expect in many regions of the brain.

Second, we then combined these signals using a random weighted average, where weights were sampled from a uniform distribution and normalized to sum to one. These voxel weights were stable across runs within a subject but differed between subjects. We next convolved the neural time series of each voxel with the species-specific canonical HRF (MION or BOLD). The resulting voxel time series were then z-scored.

Finally, we compared four different methods to account for the temporal misalignment introduced by the different HRFs:

1) Double convolution. Following Mantini et al. (2012b, 2012a) we convolve each species' data with the other species' canonical HRF, placing both time series in an intermediate' temporal space. This approach enables voxel-wise comparisons but imposes strong low-pass filtering. Reliance on canonical HRFs is also subject to compounded HRF mis-modeling error, which we do not consider here.
2) Multiview without temporal alignment. We fit a variance-partitioning multiview model (the Shared Response Model; Chen et al., 2015) directly to untransformed BOLD and MION data, projecting view-specific data into a shared latent space. This provides a baseline test of the degree to which multiview representational alignment can recover cross-species structure without correcting the temporal mismatch between MION and BOLD.
3) Temporal alignment without multiview. We learned a convolutional kernel that maps MION-to-BOLD by minimizing a voxel-space mismatch objective (also sum of squared

bioRxiv preprint doi: https://doi.org/10.64898/2026.10.01.755966; this version posted October 2, 2026. The copyright holder for this preprint (which was not certified by peer review) is the author/funder, who has granted bioRxiv a license to display the preprint in perpetuity. It is made available under a CC-BY 4.0 International license.

errors) between MION and BOLD voxel timeseries. This approach tests whether a data-driven temporal transform can be recovered without partitioning unshared variance.

4) MVTA.

Together, these methods form an ablation grid designed to isolate the core methodological factors of interest: canonical versus learned temporal alignment, and the absence versus presence of multiview variance partitioning.

Method	Temporal Alignment	Variance Partitioning
1. Double convolution	Canonical HRFs	No
2. Multiview without temporal alignment	None	Yes
3. Temporal alignment without multiview	Learned	No
4. MVTA	Learned	Yes

We evaluated these methods in two ways. First, we evaluated temporal alignment on 10,000 held-out neural time series. For each, we compared the BOLD-convolved signal with the corresponding signal produced by each alignment procedure; for the no-alignment condition, this was the MION-convolved signal. Performance was measured by correlation with BOLD (Supplementary Figure 1A). Second, to estimate shared stimulus information, we performed Stimulus Epoch Decoding (SED) in the SRM-derived common space representation of the held out stimulus (Supplementary Figure 1B).

The two analyses yielded similar results—MVTA-corrected MION timeseries performed best, strongly outperforming alternatives. Applying the multiview approach without any temporal alignment performed reasonably well (as the SRM can accommodate the difference in the sign of the HRFs; Figure 1D). We caution against using this approach, however, as differences in the timing of the peak HRF signal would introduce systematic temporal bias. Delayed neural responses measured with the earlier-peaking MION signal would spuriously appear more like earlier responses measured with the slower-peaking BOLD signal.

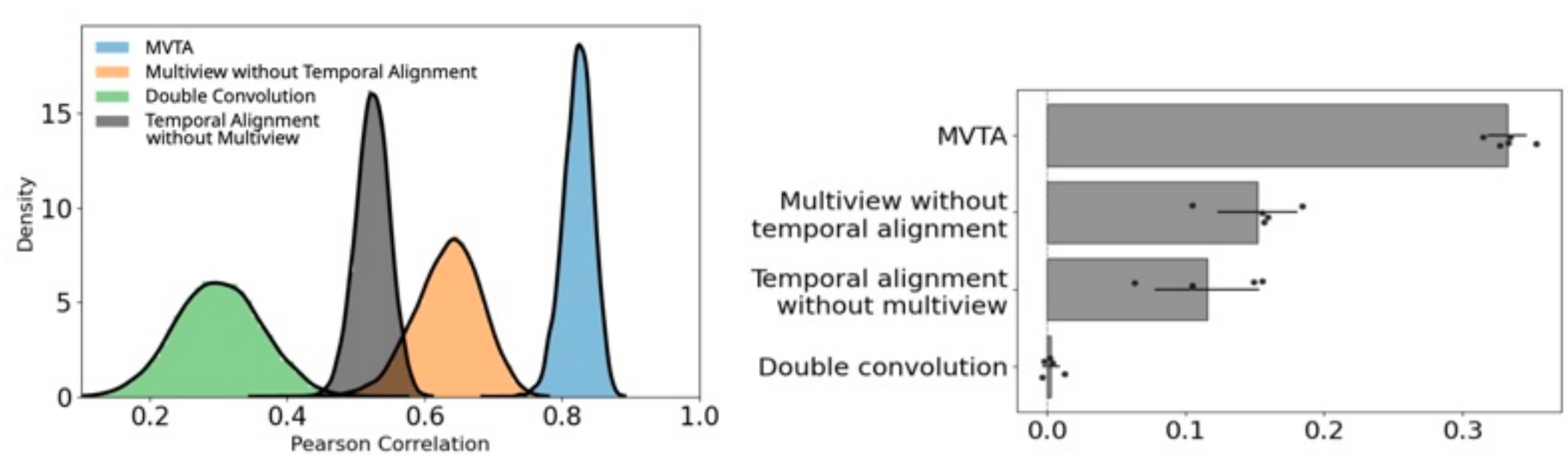

*Supplementary Figure 1. **A)**: Distribution of correlations between ground-truth BOLD and transformed MION signals across 10,000 held-out simulated neural time courses for each learned HRF method. MVTA (blue, mean r=0.82) substantially outperformed multiview without temporal alignment (orange, mean r=0.63), temporal alignment without multiview (black, mean r=0.52), and double convolution (green, mean r=0.3).Black lines depict kernel density estimates of the underlying histograms. **B)**: Shared stimulus information (SED) between BOLD and transformed MION in shared space. Mean accuracy across folds (error bars: +- 1SD; points: individual folds). MVTA substantially outperformed alternative approaches.*

bioRxiv preprint doi: https://doi.org/10.64898/2026.10.01.755966; this version posted October 2, 2026. The copyright holder for this preprint (which was not certified by peer review) is the author/funder, who has granted bioRxiv a license to display the preprint in perpetuity. It is made available under a CC-BY 4.0 International license.

Hemodynamic Transform Algorithm (MVTA). An HRF transform was learned for each between-species ROI pair through a multiview approach using multivoxel movie and temporally synchronized x static image data. To promote generalization and prevent overfitting, the transform was trained and evaluated using nested cross-validation. We used a fixed shared space dimensionality of 50. In principle, individual subject transforms could be learned, but we opted to learn a single transform for each ROI pair to reduce computational cost while accounting for differences between species and brain-regions. Learning a transform from BOLD to MION would be more complicated due to its later signal onset. Code demonstrating the algorithm on simulated movie responses is available at https://github.com/kbraunlich/multiview_temporal_alignment.

Because the MION response typically precedes the onset of the BOLD response, learning the reverse transformation (BOLD to MION) is fundamentally more difficult as it requires predicting the MION signal that occurs prior to BOLD onset. In cases where it is unknown which modality's temporal filter responds first, the mapping can simply be fit in both directions on training data, allowing the framework to empirically select the path that yields the best temporal alignment.

1. **Outer loop cross-validation**: Divide the training set stimuli into K folds. For each stimulus k_i in the K folds:
   a. Assign stimulus k_i to the outer fold test set, and the others to the outer fold training set.
   b. Create an informed guess for the transformation from MION to BOLD[5]
   i. Estimate a fixed SRM dimensionality using the outer folder training data only. Transform MION data using the canonical MION-to-BOLD transform, fit a probe SRM. Optionally compute the fractional temporal energy of each shared component and retain components with at least the uniform expected share of energy (1/K; at least one component is selected).

   c. **Inner loop cross-validation**: Divide the outer-fold training set stimuli into $K–1$ folds. For each k_j in the $K–1$ folds:
   i. Convolve outer loop training set MION data with the proposed transform.
   ii. Assign stimulus k_j to the *inner loop* test set, and the others to the *inner loop* training set:
   – Fit multiview model using the fixed SRM dimensionality on *inner loop* training set.
   – Transform *inner loop* test set data to shared space.
   – Calculate the average sum of squared errors between the BOLD and MION *inner loop* test set shared spaces.
   d. Repeat Step 1c, each time modifying the proposed transformation to minimize the average between-group disparity, until convergence (using BFGS).
   e. Apply the learned transformation to outer-loop training and test MION (kernel estimated without the test stimulus).
   f. Hyperalignment is fit on the outer-loop training stimuli and then used to transform the held-out test stimulus into the shared space; SED is computed on this test-set projection.

2. Calculate average movie segment decoding accuracy across K outer loop folds.

---

[5] We used the transform learned from simulated movie data using canonical HRFs (like that depicted in Figure 1D, main text, but with 2.5s TR).

bioRxiv preprint doi: https://doi.org/10.64898/2026.10.01.755966; this version posted October 2, 2026. The copyright holder for this preprint (which was not certified by peer review) is the author/funder, who has granted bioRxiv a license to display the preprint in perpetuity. It is made available under a CC-BY 4.0 International license.

Temporal Differences across V1-V4 and MT. In Supplementary Figure 2, we plot the MVTA-derived transforms between macaque V1, and human V1—V4 + MT. Relative to the transforms involving macaque V1-V4 (which are highly similar), the transform involving macaque MT is delayed in time. Although this is consistent with MT's known role in motion processing, this possibility cannot be distinguished from a regional difference in hemodynamic response shape.

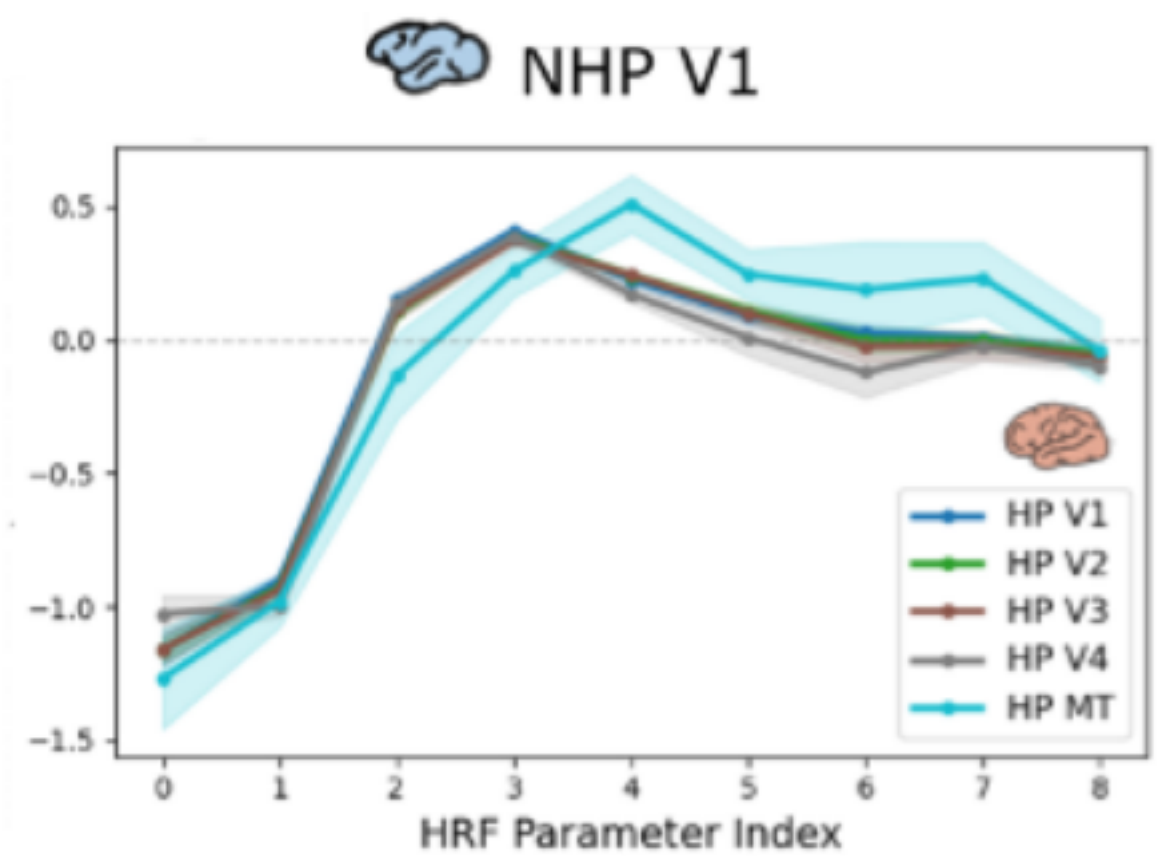

Supplementary Figure 2. Learned HRF transform between pRF-defined macaque V1 and human V1, V2, V3, V4, and MT (TR = 2.5 s). *Shaded regions indicate ±1 SD across runs (three movies and two static image sequences). The learned transform was largely consistent from macaque V1 to human V1–V4, whereas the transform to human MT showed a more gradual rise and a later peak. It is unclear whether this difference reflects neural timing or regional variation in the HRF.*

Cross-Species Dorsal and Ventral Components of V1-V4. Retinotopic visual areas V1, V2, and V3 are organized into separate representations of the upper and lower visual fields (the extent to which this is also true for V4 is debated; Goddard et al., 2011; Hansen et al., 2007; Wade et al., 2008; Winawer et al., 2010). Here we ask: Does cross-species correspondence preserves the upper-lower visual field representational structure? We split each pRF-derived ROIs into their dorsal and ventral components and repeated the between-species analyses (Supplementary Figure 3). After calculating shared stimulus information (SED) values for all ROI-pairs, we fit regression models to each subject. Group-level inference was then performed on the resulting subject-wise regression coefficients using one-sample *t*-tests and Wilcoxon signed-rank tests. As with the full pRF-derived ROIs, we included terms for stimulus (movies vs. static images), hierarchical bias (upper vs. lower heatmap triangle), representational fingerprints (diagonal vs. off-diagonal), and canonical hierarchical structure. However, we additionally included a regressor for dorsal/ventral asymmetry (matched vs. mismatched dorsal ventral components). Finally, we included all two-way interactions with stimulus type. Although we include human V4 in the heatmaps of Supplementary Figure 3, we exclude it from statistical analyses due to uncertainty about the relative organization of its dorsal component between species (Goddard et al., 2011; Hansen et al., 2007; Wade et al., 2008; Winawer et al., 2010).

bioRxiv preprint doi: https://doi.org/10.64898/2026.10.01.755966; this version posted October 2, 2026. The copyright holder for this preprint (which was not certified by peer review) is the author/funder, who has granted bioRxiv a license to display the preprint in perpetuity. It is made available under a CC-BY 4.0 International license.

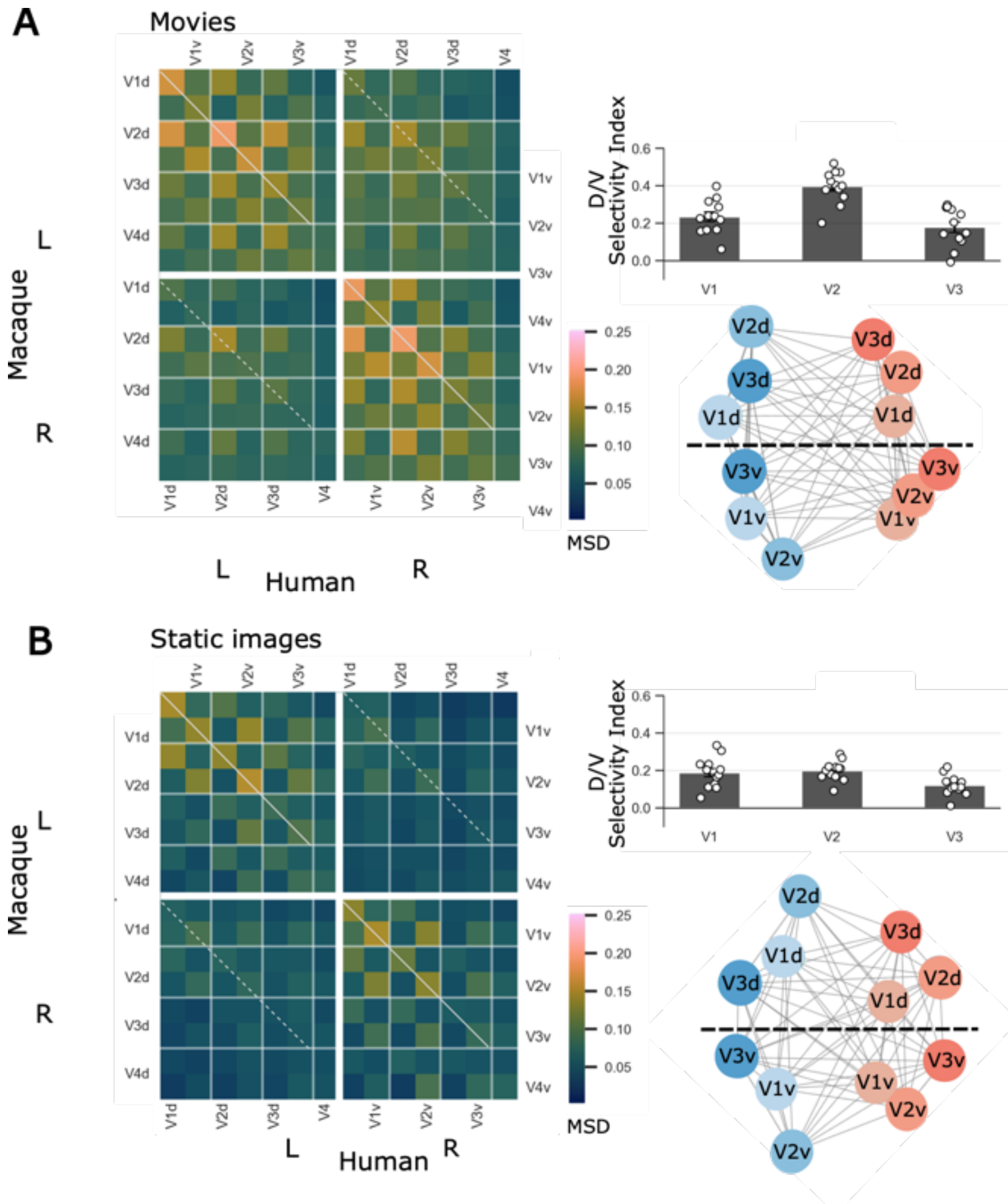

Supplementary Figure 3. ***(A)*** *Movie viewing (left): Heatmaps of cross-species SED during naturalistic movie viewing. Representational similarity is stronger between matched dorsal and ventral subdivisions. The matrices display the hierarchical asymmetry effect—where higher-order macaque regions share more information with lower-order human regions (e.g., macaque V3/V4 sharing high similarity with human V1/V2). A hemisphere bias is also present: SED between macaque V3/V4 and human V1–V3 is greater for the left-macaque/right-human pairing than for the reverse. White lines depict the identity diagonal (solid = matched hemispheres; dashed = mismatched hemispheres). Dorsal/ventral selectivity index* ***(top right):*** *Mean dorsal/ventral (d/v) selectivity index across participants for visual areas V1, V2, and V3 (V4 is excluded). For each participant and visual area, four SED values were extracted from the movie-viewing cross-species matrix by first averaging over matched hemispheres (left-to-left and right-to-right), then averaging over the dorsal and ventral components within each subdivision category: matched = mean of dorsal-to-dorsal and ventral-to-ventral; mismatched = mean of dorsal-to-ventral and ventral-to-dorsal. The d/v selectivity index was defined as (match − mismatch) / (match + mismatch). An index of zero indicates no subdivision preference; positive values indicate greater SED accuracy for subdivision-matched than subdivision-mismatched region pairs. Bars show the group mean; error bars indicate the standard error of the mean. Individual participant values are overlaid as points.* ***(B)*** *Static images (left): Heatmaps of cross-species SED during static image viewing. Under these static stimulus conditions, the hierarchical asymmetry effect is not apparent. Multidimensional* ***scaling (bottom right):*** *Joint within- and cross-species MDS space. The two dominant dimensions primarily capture species differences (horizontal axis) and dorsal-ventral representational differences (vertical axis), as these binary classes are linearly separable. Although the hierarchical organization is somewhat obscured in this two-dimensional projection, it remains apparent in the full heatmaps.*

As with the full ROI analysis, we found that shared stimulus information (SED) during movies tended to be greater than during static image sequences ($t(11) = 10.04$, $p < 0.001$, $W = 0$, $p < 0.001$). The main hemispheric effect was not significant ($t(11) = 0.47$, $p = 0.65$, $W = 27$, $p =$

bioRxiv preprint doi: https://doi.org/10.64898/2026.10.01.755966; this version posted October 2, 2026. The copyright holder for this preprint (which was not certified by peer review) is the author/funder, who has granted bioRxiv a license to display the preprint in perpetuity. It is made available under a CC-BY 4.0 International license.

0.38), however, the hemispheric effect was significantly stronger during movies (M β = 0.3, SEM β = 0.05) than during static images (M β = 0.01, SEM β = 0.03) the interaction was significant ($t(11) = 6.11$, $p < 0.001$, $W = 1$, $p < 0.001$). Diagonal values (M β = 0.36, SEM β = 0.04) were significantly greater than off-diagonal values ($t(11) = 3.39$, $p < 0.001$, $W = 0$, $p < 0.001$), indicating distinct representational fingerprints. This effect did not differ between stimulus types ($t(11) = -0.62$, $p = 0.54$, $W = 33$, $p = 0.68$). Although this pattern is not readily apparent in the two-dimensional multidimensional scaling projections (Figure 1G), which provide only low-dimensional visualizations of the full distance structure, the off-diagonal values (M β = 0.17, SEM β = 0.016 , $t(11) = 10.6$, $p < 0.001$, $W = 0$, $p < 0.001$), and this effect was not significantly different between movies and static image stimuli ($t(11) = 2.02$, $p = 0.07$, $W = 16$, $p = 0.08$). SED tended to be stronger for matched dorsal/ventral components than mismatched components (M β = 0.46, SEM β = 0.046, $t(11) = 9.91$, $p < 0.001$, $W = 0$, $p < 0.001$), and this effect did not differ between stimulus types ($t(11) = 0.34$, $p = 0.7$, $W = 35$, $p = 0.8$).

The two MDS axes discriminate species and the dorsal/ventral split.

Methodological Considerations and Contributions. A practical consideration concerns behavioral training requirements. While we trained the first 2 NHPs (Ri and Mo) to perform a 1-back task during the localizer tasks and the static image stimulus, we subsequently added a 3rd macaque subject (Wo) without the behavioral task. We found that results largely generalized to this macaque, suggesting that (at least for the brain regions identified here) behavioral task is not strictly necessary for reliable localization and identification of shared signal.

bioRxiv preprint doi: https://doi.org/10.64898/2026.10.01.755966; this version posted October 2, 2026. The copyright holder for this preprint (which was not certified by peer review) is the author/funder, who has granted bioRxiv a license to display the preprint in perpetuity. It is made available under a CC-BY 4.0 International license.

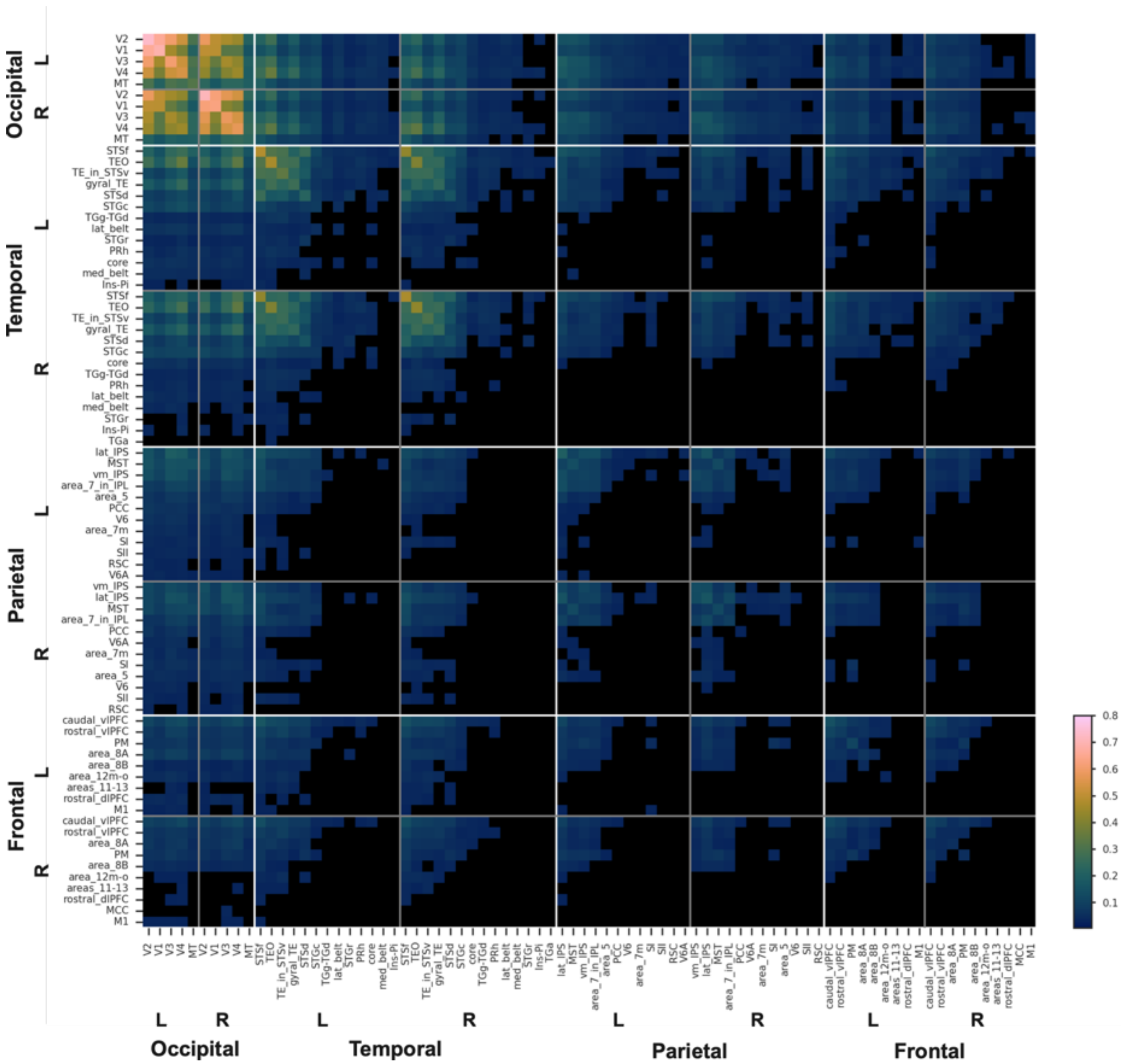

Supplementary Figure 4. Macaque CHARM (4) atlas average SED accuracy across all stimuli. (FDR corrected mean binomial test q<0.05 and all subject SED >chance.)

bioRxiv preprint doi: https://doi.org/10.64898/2026.10.01.755966; this version posted October 2, 2026. The copyright holder for this preprint (which was not certified by peer review) is the author/funder, who has granted bioRxiv a license to display the preprint in perpetuity. It is made available under a CC-BY 4.0 International license.

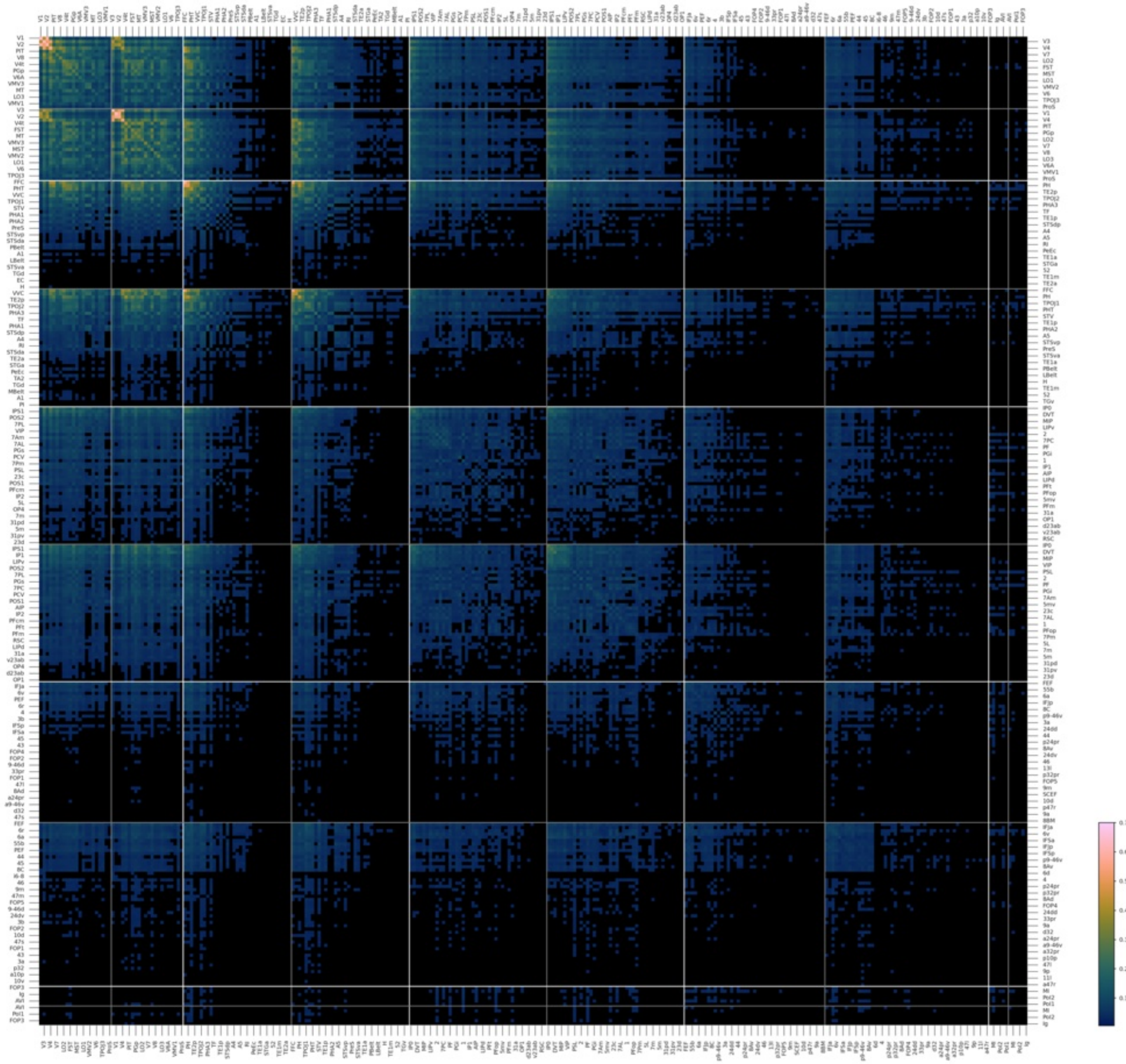

Supplementary Figure 5. Human Glasser atlas average within-HP SED accuracy across all stimuli. Note that odd and even numbered labels alternate between top/bottom and left/right axes. (FDR corrected mean binomial test q<0.05 and all subjects>chance.)

Human Brain Coverage

bioRxiv preprint doi: https://doi.org/10.64898/2026.10.01.755966; this version posted October 2, 2026. The copyright holder for this preprint (which was not certified by peer review) is the author/funder, who has granted bioRxiv a license to display the preprint in perpetuity. It is made available under a CC-BY 4.0 International license.

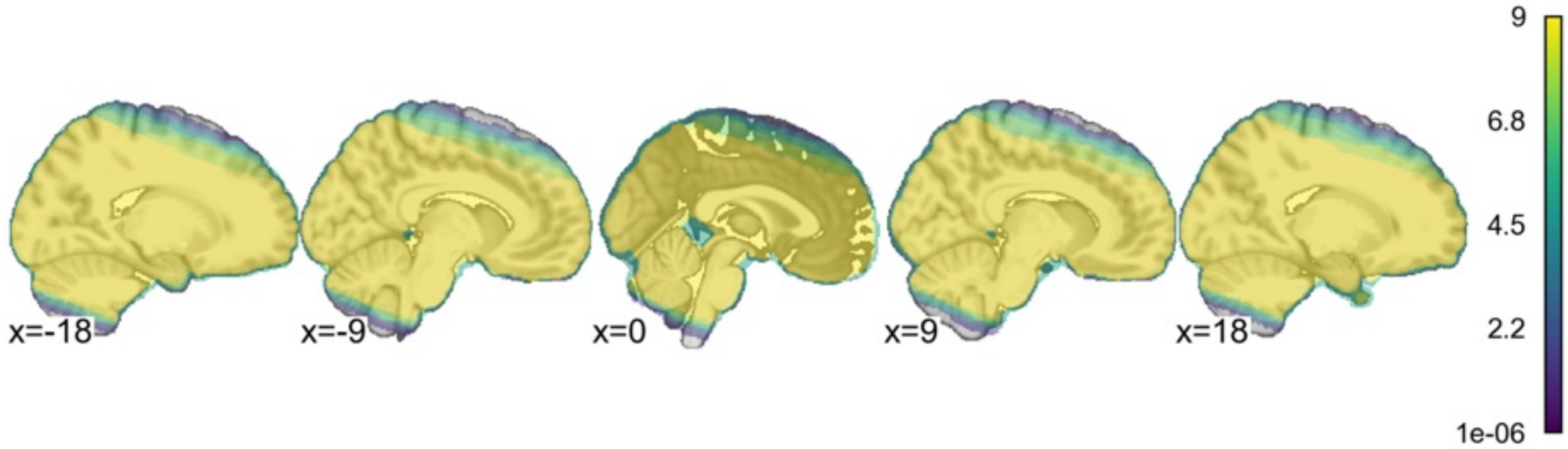

Supplementary Figure 6. Human brain coverage. Colormap indicates the number of subjects with coverage at each voxel.

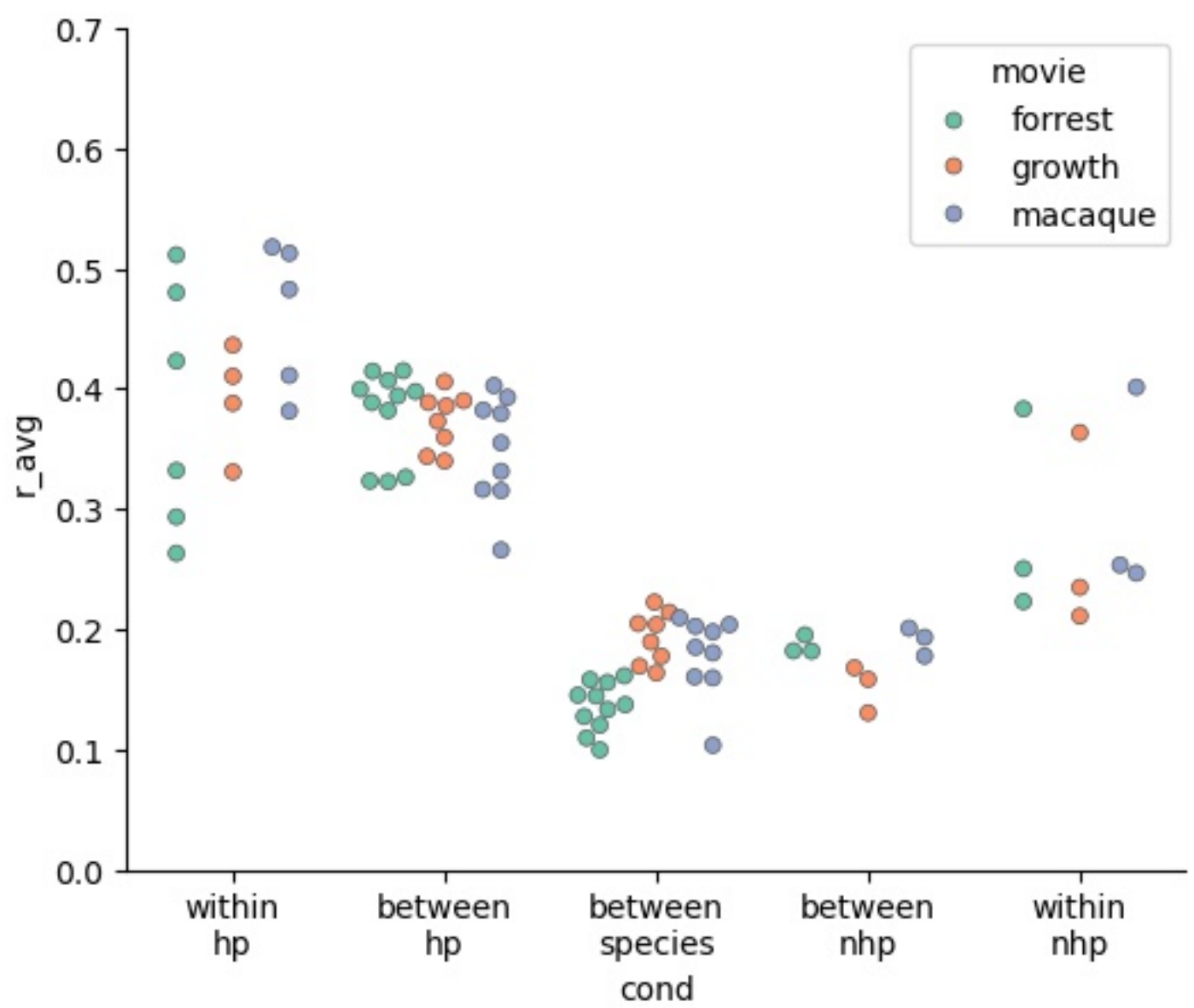

Supplementary Figure7 Pearson correlation coefficient between eye-movement traces. Within HP and NHP are reliability within individual subjects. Between HP and NHP are reliability across subjects within each species. Between species is across species. Not all HP subjects had reliable eye-tracking data for all stimuli.

bioRxiv preprint doi: https://doi.org/10.64898/2026.10.01.755966; this version posted October 2, 2026. The copyright holder for this preprint (which was not certified by peer review) is the author/funder, who has granted bioRxiv a license to display the preprint in perpetuity. It is made available under a CC-BY 4.0 International license.

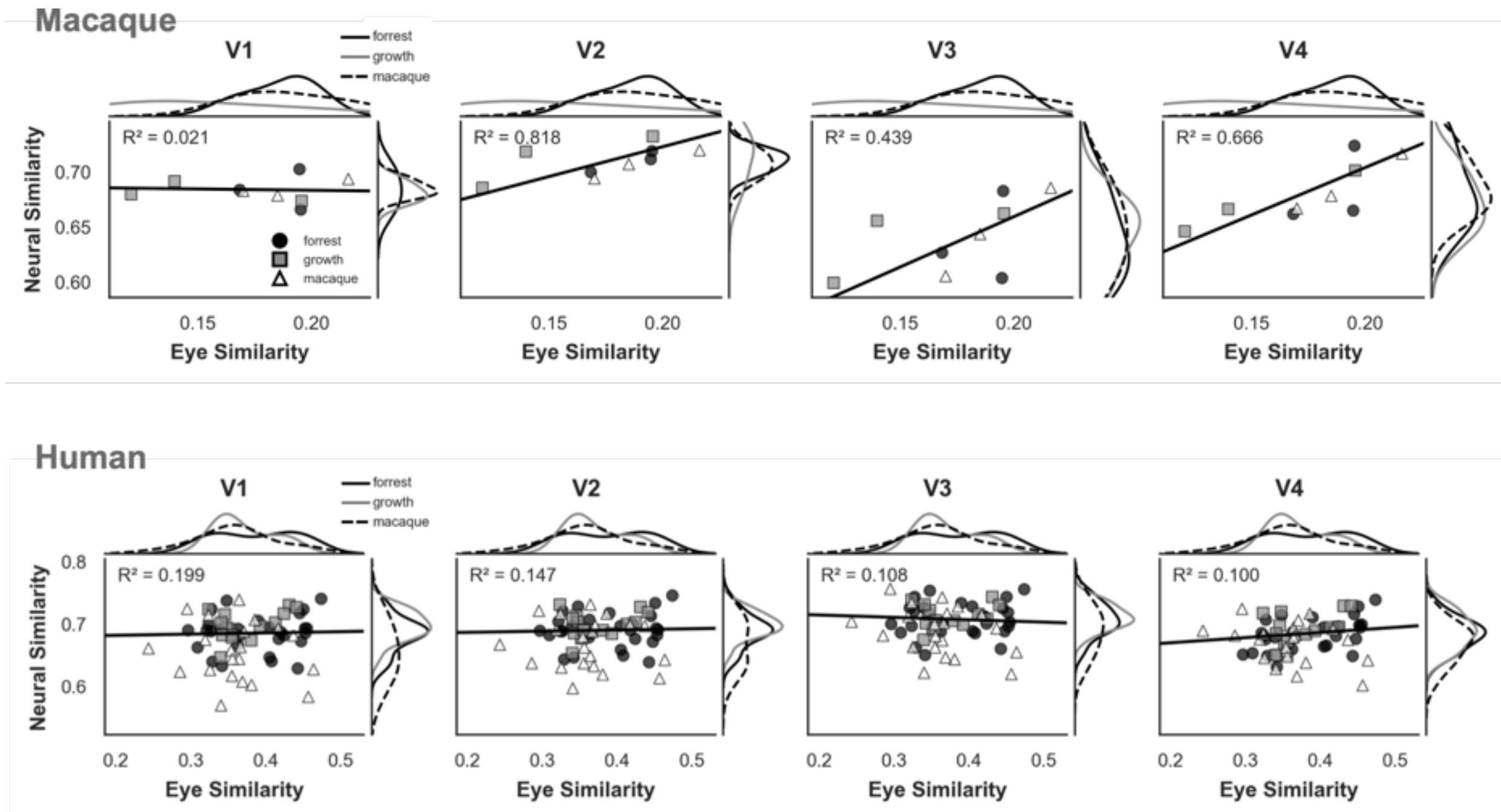

Supplementary Figure 8. Within-species relationship between eye-movement similarity and neural similarity in early visual cortex. Scatter plots show eye (Pearson correlation) and neural similarity (Spearman correlation) for each human-macaque subject pair. Marginal distributions depict the corresponding similarity values. Filled circles: Forrest Gump; filled squares: Growth; open triangles: Macaque clips. Black lines indicate least-squares regression slopes in the original units (unstandardized). Positive slopes indicate that subject pairs with more similar eye movements exhibit more similar neural responses. Because of three statistical challenges, we treat these analyses as descriptive and do not estimate statistical significance. 1) As each subject contributes to multiple ROI pairs, they are not independent. 2) With N=3, the macaques have an insufficient number of pairs. 3) In humans, the uniformly high similarity of eye-movement traces limits sensitivity to an association with neural similarity.

Supplementary Table 1: Regions with high expansion and high information sharing (negative contribution to the global correlation). Although regions with high cross-species information sharing tended to localize to areas associated with low cortical expansion, several regions showed high cross-species information sharing despite substantial cortical expansion. These regions may reflect strong selective pressure to preserve function during cortical expansion or, alternatively, an increased allocation of cortical resources to support those functions.

label	n_vertices	mean_contrbution	mean_SED	mean_expansion_z
R_TPOJ1	1639	-0.682605	0.081291	0.995697
R_TE2p	862	-0.496862	0.068781	1.05702
R_LIPv	661	-0.465371	0.062715	1.273024
R_TPOJ2	827	-0.464787	0.076752	0.765267
R_PHT	759	-0.358675	0.061059	1.064206

bioRxiv preprint doi: https://doi.org/10.64898/2026.10.01.755966; this version posted October 2, 2026. The copyright holder for this preprint (which was not certified by peer review) is the author/funder, who has granted bioRxiv a license to display the preprint in perpetuity. It is made available under a CC-BY 4.0 International license.

R_STV	894	-0.319639	0.061587	0.923461
R_MST	385	-0.156272	0.108016	0.136374
R_TE1p	1157	-0.14991	0.04818	1.301607
R_TPOJ3	532	-0.148761	0.078149	0.235596
R_FST	401	-0.106974	0.101519	0.103457
R_IP1	1019	-0.078674	0.051782	0.443907
R_TF	1370	-0.035853	0.048529	0.295859
R_FEF	850	-0.029756	0.047562	0.284671
R_MIP	815	-0.002675	0.047261	0.026925
